# Structural Expansion of Chalcogenido Tetrelates in Ionic Liquids by Incorporation of Sulfido Antimonate Units

**DOI:** 10.1002/chem.202003887

**Published:** 2020-10-14

**Authors:** Bertram Peters, Chloé Krampe, Julian Klärner, Stefanie Dehnen

**Affiliations:** ^1^ Fachbereich Chemie und Wissenschaftliches Zentrum für, Materialwissenschaften (WZMW) Philipps-Universität Marburg Hans-Meerwein-Straße 4 35043 Marburg Germany

**Keywords:** antimony, germanium, sulfido (semi)metalates, tin, X-ray diffraction

## Abstract

Multinary chalcogenido (semi)metalate salts exhibit finely tunable optical properties based on the combination of metal and chalcogenide ions in their polyanionic substructure. Here, we present the structural expansion of chalcogenido germanate(IV) or stannate(IV) architectures with Sb^III^, which clearly affects the vibrational and optical absorption properties of the solid compounds. For the synthesis of the title compounds, [K_4_(H_2_O)_4_][Ge_4_S_10_] or [K_4_(H_2_O)_4_][SnS_4_] were reacted with SbCl_3_ under ionothermal conditions in imidazolium‐based ionic liquids. Salt metathesis at relatively low temperatures (120 °C or 150 °C) enabled the incorporation of (formally) Sb^3+^ ions into the anionic substructure of the precursors, and their modification to form (Cat)_16_[Ge_2_Sb_2_S_7_]_6_[GeS_4_] (**1**) and (Cat)_6_[Sn_10_O_4_S_20_][Sb_3_S_4_]_2_ (**2 a** and **2 b**), wherein Cat=(C_4_C_1_C_1_Im)^+^ (**1** and **2 a**) or (C_4_C_1_C_2_Im)^+^ (**2 b**). In **1**, germanium and antimony atoms are combined to form a rare noradamantane‐type ternary molecular anion, six of which surround an {GeS_4_} unit in a highly symmetric secondary structure, and finally crystallize in a diamond‐like superstructure. In **2**, supertetrahedral oxo‐sulfido stannate clusters are generated, as known from the ionothermal treatment of the stannate precursor alone, yet, linked here into unprecedented one‐dimensional strands with {Sb_3_S_4_} units as linkers. We discuss the single‐crystal structures of these uncommon salts of ternary and quaternary chalcogenido (semi)metalate anions, as well as their Raman and UV‐visible spectra.

## Graphical abstract

**Three sulfido (semi)metalates**, (Cat)_16_[Ge_2_Sb_2_S_7_]_6_[GeS_4_] (**1**) and (Cat)_6_[Sn_10_O_4_S_20_][Sb_3_S_4_]_2_ (**2 a** and **2 b**), Cat=(C_4_C_1_C_1_Im)^+^ (**1** and **2 a**) or (C_4_C_1_C_2_Im)^+^ (**2 b**), were obtained by salt metathesis from [K_4_(H_2_O)_4_][Ge_4_S_10_] or [K_4_(H_2_O)_4_][SnS_4_] and SbCl_3_ in imidazolium‐based ionic liquids at moderate temperatures (120 °C). The incorporation of Sb^3+^ ions into the (modified) anionic substructures is reflected in the Raman and UV‐visible spectra of the products.

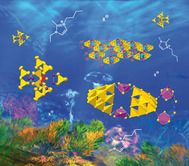

## Introduction

Metal chalcogenide compounds possess a variety of desirable properties, mostly based on their intrinsic semiconducting, photo‐conducting or ion conducting properties.[^1^, ^2^, ^3^, ^4^, ^5^, ^6^, ^7^, ^8^, ^9^] However, beside the native features of corresponding binary M_x_E_y_ compounds (M=*d*‐block or *p*‐block (semi)metal, E=O, S, Se, or Te), the combination of different M or different E atoms, and the formation of chalcogenido (semi)metalates by (formal) incorporation of Cat_2_E (Cat=alkali metal or non‐metal cation) can be used to additionally tune the corresponding structural and electronic properties.[^10^, ^11^, ^12^, ^13^, ^14^, ^15^]

In order to achieve a targeted synthesis of such compounds, various synthetic methods have been applied.[^16^, ^17^, ^18^, ^19^, ^20^, ^21^] Approaches at moderate temperatures were particularly successful in the construction of a large diversity of architectures that would not be accessed with traditional solid state methods at high temperatures. Besides comparably established syntheses under solvothermal reaction conditions[^16^, ^17^, ^18^, ^19^, ^20^] or in alkali metal polychalcogenide flux,^[21]^ reactions in ionic liquids have become a very popular method in more recent times for the transformation of chalcogenido (semi)metalates. The products are usually obtained as single‐crystalline material, which allows the subsequent investigation of their structural as well as concomitant physical properties.[^20^, ^22^, ^23^, ^24^, ^25^, ^26^, ^27^, ^28^, ^29^]

In our work, we have been particularly interested in the formation and transformation of chalcogenido tetrelate anions [Tt_x_E_y_]^q−^ (Tt=Ge, Sn), thereby addressing changes of structures and physical properties in a threefold manner, by a) dimensional changes within binary networks,[^25^, ^30^, ^31^, ^32^, ^33^, ^34^] b) combination of two types of tetrel atoms,[^35^, ^36^] and c) admixture of transition metal atoms.[^34^, ^37^] In all three cases, the whole reaction space—including the nature of the ionic liquid, the used precursors, potential auxiliary compounds, reaction temperature and time—needs to be scanned to get a sufficiently deep insight into the corresponding reaction system. As a side effect, we could recently show that alkylimidazolium‐based ionic liquids (C_l_C_m_C_n_Im)[An] (C_l_, C_m_, C_n_=alkyl groups with l, m, or n C atoms in 1, 2, or 3 position of the imidazolium (Im) ring; [An]=Cl, Br, [BF_4_]) can serve as mild and relatively benign alkylation reagents for these weakly nucleophilic chalcogenido (semi)metalate compounds, which has also been the only way of achieving their post‐synthetic alkylation to date.^[37]^

As a new means of tuning the chalcogenido (semi)metalates’ structural and electronic properties, we intended to mix two main group (semi)metal atoms of different main groups in corresponding networks. This was achieved by reacting salts of tetrahedral sulfido germanate or stannate anions, [K_4_(H_2_O)_4_][Ge_4_S_10_] or [K_4_(H_2_O)_4_][SnS_4_],^[38]^ with SbCl_3_ in imidazolium‐based ionic liquids (C_4_C_1_C_1_Im)[An]. Instead of a mere cation exchange, potentially along with a reorganization of the anionic substructure (as observed at the formation of 1D‐(C_4_C_1_Im)_2_[Ge_4_Se_9_]^[33]^ or (Cat)_4_[Sn_10_O_4_S_16_(SMe)_4_] with Cat=C_4_C_1_C_1_Im,^[37]^ for instance, which form in the absence of other metal compounds), the pnictogen atoms were incorporated in the structures under ionothermal conditions. This way of combining tin sulfide and antimony sulfide subunits has been unprecedented. It should be noted that another compound with antimony sulfide subunits that was prepared in ionic liquids, [Sb_7_S_8_Br_2_](AlCl_4_)_3_, was synthesized from the elements in an acidic ionic liquid mixture.^[39]^ As a result, the clusters in this very interesting compound bearing non‐linear optical properties are cationic, and thus differ significantly from the compounds presented in this work.

Here, we report about the synthesis, structures, and optical absorption properties of the products of the mentioned reactions, (Cat)_16_[Ge_2_Sb_2_S_7_]_6_[GeS_4_] (**1**) and (Cat)_6_[Sn_10_O_4_S_20_][Sb_3_S_4_]_2_ (**2 a** and **2 b**), wherein Cat=(C_4_C_1_C_1_Im)^+^ (**1** and **2 a**) or (C_4_C_1_C_2_Im)^+^ (**2 b**).

## Results and Discussion

**Scheme 1:**
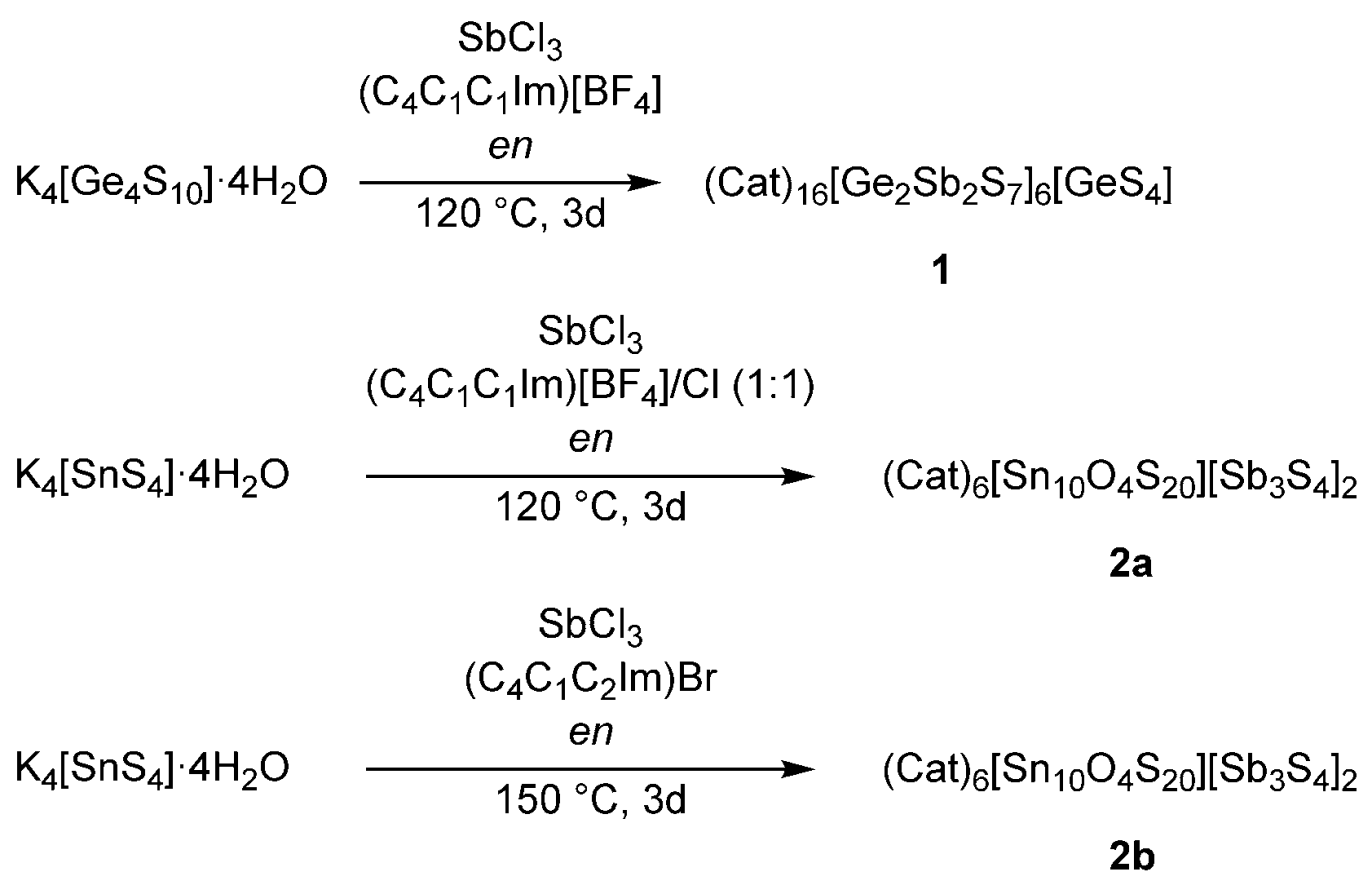
Overview of the synthesis of (Cat)_16_[Ge_2_Sb_2_S_7_]_6_[GeS_4_] (**1**) and (Cat)_6_[Sn_10_O_4_S_20_][Sb_3_S_4_]_2_ (**2**). The cations of compounds **1** and **2 a** are heavily disordered in the crystal structures and could therefore not be determined by means of single‐crystal X‐ray diffraction, yet most probably, ‘Cat’ represents tris‐alkylated imidazolium cations (C_4_C_1_C_1_Im)^+^, according to single‐crystal Raman spectroscopy (see below). This is indirectly supported by the absence of considerable amounts of K according to micro X‐ray fluorescence (μ‐XRF) spectroscopy (see the Supporting Information) and by the presence of crystallographically determined (C_4_C_1_C_2_Im)^+^ cations in **2 b** (with ‘C_4_’, ‘C_1_’, and ‘C_2_’ specifying the chain lengths of the butyl, methyl, and ethyl substituents of the imidazolium ring denoted as ‘Im’). By‐products (potassium halides and tetrafluoridoborate) are not indicated here; *en*=ethane‐1,2‐diamine.

The synthesis of **1** and **2** proceeded according to Scheme 1, by ionothermal treatment of [K_4_(H_2_O)_4_][Ge_4_S_10_] or [K_4_(H_2_O)_4_][SnS_4_] with SbCl_3_ in the ionic liquid (C_4_C_1_C_1_Im)[BF_4_], in the ionic liquid mixture (C_4_C_1_C_1_Im)[BF_4_]/(C_4_C_1_C_1_Im)Cl (1:1), or in (C_4_C_1_C_1_Im)Br, respectively. The structures of the single‐crystalline compounds were determined by means of single‐crystal X‐ray diffraction.^[40]^

**Figure 1:**
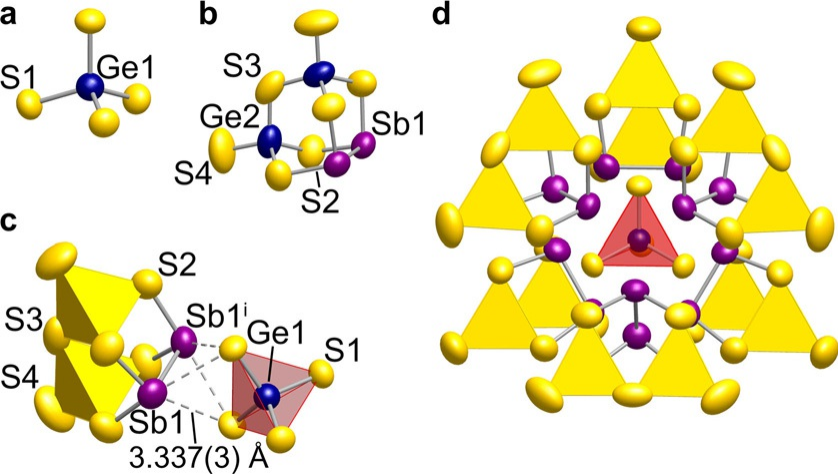
Subunits within the anionic substructure of compound **1**. a) Tetrahedral anion [GeS_4_]^4−^. b) Noradamantane‐type anion [Ge_2_Sb_2_S_7_]^2−^. c) Relative orientation of the two types of anions in crystal structure of **1**. d) Structure of the resulting supramolecular subunit 0∞ {([Ge_2_Sb_2_S_7_]_6_[GeS_4_])^16−^}, with the {GeS_4_} tetrahedra in the two anions highlighted as red (Ge1) and yellow (Ge2) tetrahedra, respectively. Displacement ellipsoids are drawn at a 50 % probability level. Symmetry code i: *z*, 3/4 −*x*, 3/4 −*y*.

Compound **1** crystallizes as yellow blocks in the cubic crystal system, space group *Fd* $\bar{3}$ *m*, with eight formula units in the unit cell. The structure contains two different types of anions, a tetrahedral [GeS_4_]^4−^ anion and a noradamantane‐type [Ge_2_Sb_2_S_7_]^2−^ moiety in a 1:6 ratio. Within the crystal, six [Ge_2_Sb_2_S_7_]^2−^ units surround a central [GeS_4_]^4−^ anion in a highly symmetric supramolecular assembly 0∞ {([Ge_2_Sb_2_S_7_]_6_[GeS_4_])^16−^} consisting of seven chalcogenido (semi)metalate anions in sum (Figure 1).

Each of the Sb1−Sb1’ bonds within the [Ge_2_Sb_2_S_7_]^2−^ anions are located above one of the six S⋅⋅⋅S edges of the [GeS_4_]^4−^ tetrahedra, at an S1⋅⋅⋅Sb1 distance of 3.337(3) Å, and a distance of the center of the Sb1−Sb1’ bond from the center of the S⋅⋅⋅S edges of the central tetrahedron of 2.420(1) Å. The two subunits are thus not covalently bonded. In order to explore whether they might undergo notable attractive dispersive interactions, we inspected the structural data in detail.

Within the [Ge_2_Sb_2_S_7_]^2−^ anions, the terminal Ge2−S4 bond length is 2.107(5) Å, while the bonds to the bridging μ‐S atoms, Ge2−S2 and Ge2−S3_,_ are 2.216(5) and 2.226(3) Å, respectively. The Sb1−S2 and Sb1−Sb1’ bond lengths amount to 2.454(3) and 2.832(2) Å, respectively. All of these values accord well with the data reported for the [Ge_2_Sb_2_S_7_]^2−^ anion in the related, yet overall less complex compound (Me_2_NH_2_)_6_[Ge_2_Sb_2_S_7_][Ge_4_S_10_] (Ge−S_term_ 2.136(2)–2.150(2) Å, Ge−(μ‐S) 2.207(2)–2.236(2) Å, Sb−S 2.442(2)–2.530(2) Å, Sb−Sb 2.824(1) Å; see Figure 4 a).^[41]^ The only structural deviation that is notable at all concerns the terminal Ge−S bonds, which are slightly shorter in **1** (by ≈3 pm) than in the reference compound, which can be explained by a weak additional interaction between the sulfur atom and a hydrogen atom of the (Me_2_NH_2_)^+^ cations in the latter.

**Figure 2:**
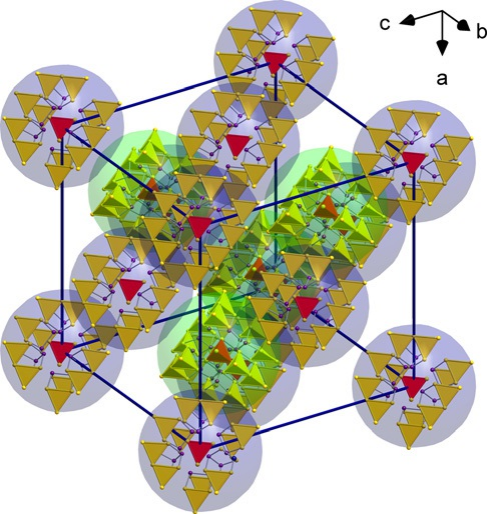
Illustration of the diamond‐like superstructure of the 0∞ {([Ge_2_Sb_2_S_7_]_6_[GeS_4_])^16−^} assemblies in compound **1**. Units that fill the positions of a cubic dense packing are enclosed in blue semi‐transparent spheres, those that occupy positions corresponding to tetrahedral holes are enclosed in green semi‐transparent spheres. These spheres have a diameter of 8.65 Å, which corresponds to the Ge1⋅⋅⋅S4 distance, and thus includes all atoms up to the outmost sulfur (S4) atoms. Note that the anions do not fill all segments of the spheres, hence allowing for a slight overlap.

The Ge1−S1 bond in the central [GeS_4_]^4−^ anion exhibits a lengths of 2.215(6) Å, in good agreement with the literature‐known crystallographic data of Mn_2_[GeS_4_] (Ge−S 2.15–2.27 Å).^[42]^ The fact that the Ge−S bonds apparently are not influenced by neighboring [Ge_2_Sb_2_S_7_]^2−^ cluster anions points at the absence of significant secondary interactions. The clustering of seven complex anions in **1** therefore seems to be driven by the nearly spherical shape of these assemblies, which are then packed in a diamond‐like superstructure in the crystal. Figure 2 illustrates the arrangement of the anions in the crystal structure of **1**.

The 0∞ {([Ge_2_Sb_2_S_7_]_6_[GeS_4_])^16−^} assemblies are separated from each other by the counterions. Although not crystallographically detectable owing to heavy disorder (which is rather common in salts obtained from ionothermal syntheses), we assume that the counterions be (C_4_C_1_C_1_Im)^+^, based on the volume of the voids between the anionic assemblies (30530.3 Å^3^ according to accessible void calculations done with PLATON).^[43]^ We note that we cannot exclude a fraction of 2–3 counterions to be K^+^, based on a small K abundance (≈4 at %) in the μ‐XRF spectrum (Figure S2, Table S1).

The ‘accessible’ void matches relatively well the volume of 16 (C_4_C_1_C_1_Im)^+^ cations per formula unit, hence 128 cations within the unit cell (≈28160 Å^3^ for an estimated 20 Å^3^ per C or N atom), given that there are additional voids between the cation molecules. Although we cannot exclude with certainty the presence of K^+^ cations as counterions between the anionic assemblies, we can exclude the presence of any cations between the [Ge_2_Sb_2_S_7_]^2−^ and [GeS_4_]^4−^ units of one such assembly, given the small interatomic distances between them (see above).

Compound **2** was obtained in two different versions, one crystallizing from (C_4_C_1_C_1_Im)[BF_4_]/Cl (1:1) as yellow blocks in the tetragonal crystal system, space group *P*4_2_/*mbc*, with four formula units per unit cell (**2 a**), and one crystallizing from (C_4_C_1_C_2_Im)Br as yellow blocks (see Figure S1) in the triclinic crystal system, space group *P* $\bar{1}$ , with two formula units per unit cell (**2 b**). While the two compounds differ in their counterions (see below), they comprise the same anionic substructure. The lower crystallographic symmetry observed for compound **2 b** results from the fact that the symmetry‐reducing counterions are localizable; hence the high symmetry of **2 a** is likely a *pseudo*‐symmetry, as the heavily disordered cations form an isotropic ‘solid solution’.

**Figure 3:**
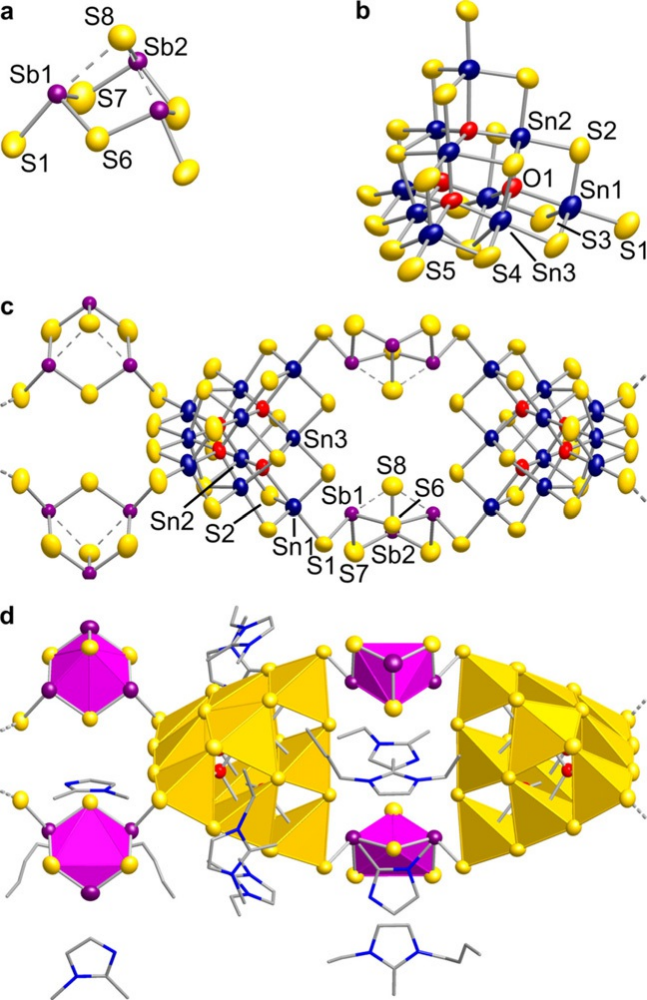
Structure of the anion in **2**. a) [Sb_3_S_6_]^3−^ unit, b) [Sn_10_O_4_S_20_]^8−^ unit, c) combination of the two former units in **2 a**, d) the same segment of the structure in **2 b**, yet with polyhedral representation and displaying the cations that were localizable in the crystal structure. Displacement ellipsoids are drawn at a 50 % probability level.

As in **1**, the anion in **2** is based on two structural motifs: a supertetrahedral [Sn_10_O_4_S_20_]^8−^ cluster (with the O atoms stemming from water residues in the ionic liquids) and a distorted defect‐heterocubane unit with two terminal sulfide ligands, [Sb_3_S_6_]^3−^. However, in contrast to **1**, the two building units are covalently linked in compound **2** to form quaternary one‐dimensional strands of the formula 1∞ {([Sn_10_O_4_S_20_][Sb_3_S_4_]_2_)^6−^} (Figure 3).

**Figure 4:**
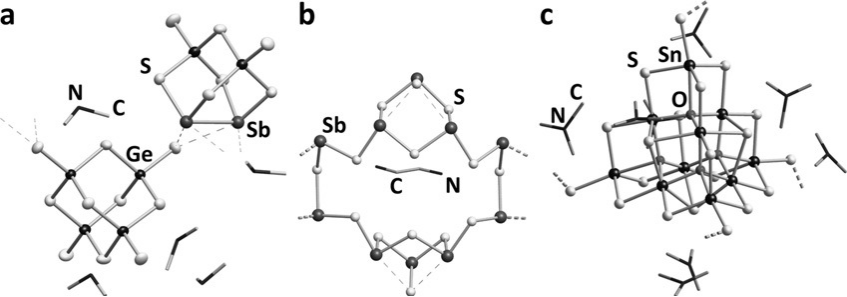
Cut‐outs from the crystal structures of known compounds that comprise structural motifs related to those in compounds **1**, **2 a**, and **2 b** reported in this work: a) (Me_2_NH_2_)_6_[(Ge_2_Sb_2_S_7_)(Ge_4_S_10_)],^[41]^ b) (C_2_H_10_N_2_)[Sb_8_S_13_],^[44]^ and c) (Me_3_NH)_4_[Sn_10_O_4_S_18_].^[45]^ Ammonium counterions are drawn in wire style.

The strands are formed by sharing all terminal sulfide (S1) atoms of both structural motifs. Thus, two atoms each on opposite edges of the supertetrahedral moiety serve as μ‐S bridges to two {Sb_3_S_4_} subunits on each side. The [Sb_3_S_6_]^3−^ moiety has been reported as a building unit in the sulfido antimonate salt (C_2_H_10_N_2_)[Sb_8_S_13_] (see Figure 4 b).^[44]^ In **2**, it is based on a central {Sb_3_(μ‐S)_3_} ring (Sb1,2−S6,7 2.445(3)–2.451(4) Å), which is capped in an unsymmetric μ_3_‐type manner by S8 (Sb2−S8 2.357(5) Å; Sb1⋅⋅⋅S8 3.092(3) Å). The length of the Sb1−S1 bond, which serves as a bridge to the supertetrahedral oxo‐sulfido stannate units, amounts to 2.446(3) Å. A comparison of the structural parameters of the anionic substructure of **2** with those reported for (C_2_H_10_N_2_)[Sb_8_S_13_] (Sb−(μ‐S) 2.468(2)–2.491(3) Å; Sb−(μ_3_‐S) 2.406(3) Å; Sb⋅⋅⋅(μ_3_‐S) 3.113(2), 3.137(2) Å; Sb−S_term_ 2.489(2)–2.496(2) Å)^[44]^ indicates slight deviations of up to ≈5 pm, with slight elongation of the Sb−(μ‐S) bonds in **2** on the one hand, and a slight shortening of the Sb−(μ_3_‐S) and Sb−S_term_ bonds in **2** on the other hand. Very obviously, this is a consequence of the different types of network structures and counterions: while ethane‐1,2‐ammonium dications connect through close H‐bonds with the anion in (C_2_H_10_N_2_)[Sb_8_S_13_], the imidazolium cations in **2 b** are found to stay farther apart. The disorder of the cations in **2 a** is another hint towards only weak interactions with the anionic network, which we take as an indirect proof for our assumption.

The [Sn_10_O_4_S_20_]^8−^ cluster anion has been known as part of a three‐dimensional network (see Figure 4 c),^[45]^ or as discrete anion, either with^[37]^ or without[^46^, ^47^] methyl groups at the terminal S atoms. In **2 a**, the cluster possesses crystallographic *S*_4_ symmetry—with only slight deviation from *T*_d_—owing to the formation of the infinite strands. One observes the following interatomic distances: Sn1−S1 2.409(3) Å, Sn1,2,3−S2,3,4 2.383(3)–2.472(3) Å, Sn−S5 2.604(3)–2.625(3) Å, Sn−O 2.074(6), Sn⋅⋅⋅O 2.504(6) Å. The reported clusters with terminal SMe groups showed very similar structural parameters (Sn−S(Me) 2.407(9) Å, Sn−(μ‐S) 2.395(10)–2.473(9) Å, Sn−(μ_3_‐S) 2.607(8)–2.650(8) Å, Sn−O and Sn⋅⋅⋅O 2.073(19)–2.552(20) Å),^[37]^ as the ‘terminal’ S atoms are actually μ‐bridging in both cases. The purely inorganic versions of the isolated supertetrahedral architecture naturally show significantly shorter Sn−S_term_ bonds (2.355–2.374 Å),[^46^, ^47^] for these S atoms indeed terminate the anion's molecular structure.

The structural motifs of the related compounds that were quoted above for comparison with the substructures of compounds **1**, **2 a**, and **2 b** are illustrated in Figure 4.

**Figure 5:**
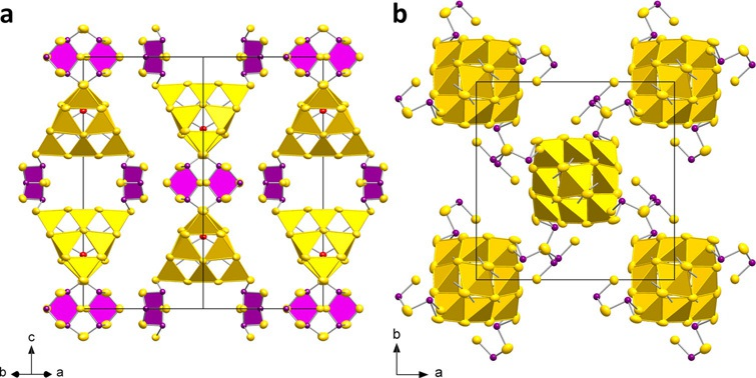
View of the crystal structure of **2** along <1,1,0> (a) and along the crystallographic *c* axis (b), shown exemplarily for **2 a**. {SnS_4_} units are shown as yellow tetrahedra, and {Sb_3_S_4_} fragments are highlighted in magenta. Displacement ellipsoids are shown at a 50 % probability level.

As the linkage of the two building units in the anionic substructure of **2** occurs via *trans*‐edges of the supertetrahedra, the entire strand possesses idealized *D*_2*h*_ symmetry (*S*_4_ in the crystal, see above), and thus, the position of the two {Sb_3_S_4_} units alternates with respect to the twofold axis (Figure 5 a). All strands run through the crystal in parallel fashion, in the direction of the crystallographic *c* axis. Neighboring strands along <0,0,1> and <1/2 ,1/2 ,1> are oriented perpendicularly to each other, which allows to optimize the positions of the {Sb_3_S_4_} groups (Figure 5).

**Figure 6:**
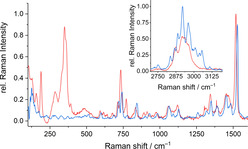
Relevant regions of the single‐crystal Raman spectra of **2 a** (red) and (C_4_C_1_C_1_Im)Cl (blue), in the range 110–1600 cm^−1^ (and 2700–3200 cm^−1^, inset).

As proven by the crystal structure of **2 b**, the cations are located between the strands, thereby forming ‘belts’ around the two alternating subunits (see Figure 3 c). In order to get an experimental hint for the actual presence of imidazolium counterions in **2 a**, we recorded Raman spectra of single‐crystals of **2 a**, and of the pure ionic liquid that was used for the reaction (Figure 6).

**Figure 7:**
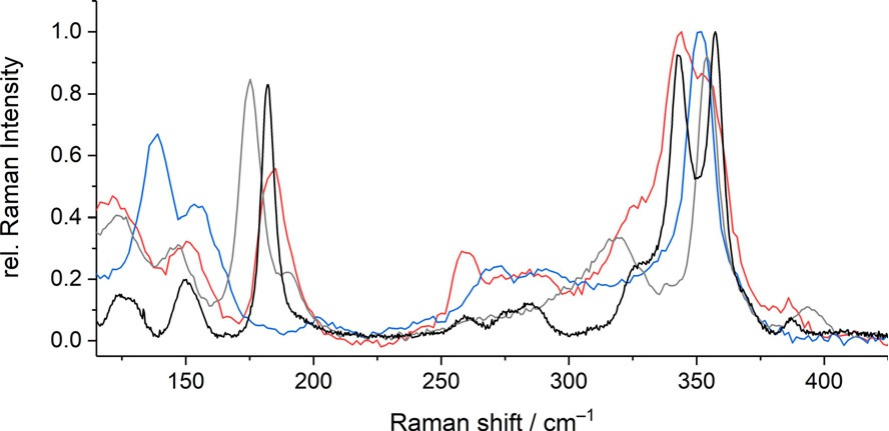
Single‐crystal Raman spectra of **1** (blue), **2 a** (red), and **2 b** (black), in comparison with the Raman spectrum reported for (Cat)_4_[Sn_10_O_4_S_16_(SMe)_4_] (grey).^[37]^

Comparison of the two Raman spectra indicate clearly the presence of the IL cation in compound **2 a**, as visible from the characteristic signals observed between 700 and 1600 cm^−1^. Another signal group which stems from C−H valence vibrations can be found between 2700 and 3200 cm^−1^ (inset in Figure 6). The signals observed at lower wavenumbers (up to 400 cm^−1^) are assigned to vibrations of the anionic substructures. These regions of the spectra of all three compounds reported herein, **1**, **2 a**, and **2 b**, and the Raman spectrum obtained from the related compound (Cat)_4_[Sn_10_O_4_S_16_(SMe)_4_],^[37]^ are presented together in Figure 7.

The spectra of **1**, **2 a** and **2 b** exhibit a broad signal consisting of two bands centered around 275 cm^−1^. As this signal is missing in the Raman spectrum of (Cat)_4_[Sn_10_O_4_S_16_(SMe)_4_],^[37]^ we attribute it to Sb−S vibrations in **1**, **2 a**, and **2 b**.

All four spectra exhibit a predominant signal at around 350 cm^−1^, which occurs as a single band in **1** and (Cat)_4_[Sn_10_O_4_S_16_(SMe)_4_] or a double band in **2 a** (shoulder) and **2 b**. As there is no structural feature that all four compounds have in common, we ascribe this signal, and another one at around 150 cm^−1^ in all spectra, to combined vibrations of the sulfido (semi)metalate substructures that are averaged around similar vibrational modes.

Some signals are only observed in the spectra of **2 a**, **2 b**, and the reference compound: a Raman signal of medium strength at ca. 125 cm^−1^, a strong band at 174–180 cm^−1^, and a signal at ca. 320 cm^−1^ in the spectrum of (Cat)_4_[Sn_10_O_4_S_16_(SMe)_4_], which in the case of **2 a** and **2 b** appears slightly blue shifted (ca. 325 cm^−1^) as a shoulder in the slightly red‐shifted, strongest signal centered around 345 cm^−1^. As said signals are missing in the spectrum of **1**, they likely stem from Sn−S and Sn−O vibrations within the supertetrahedral oxo‐sulfido stannate substructure.

A strong signal that is found exclusively in the Raman spectrum of compound **1** (ca. 140 cm^−1^), in turn, can be unambiguously assigned to Ge−S or Sb−Sb vibrations of the [Ge_2_Sb_2_S_7_]^2−^ and [GeS_4_]^4−^ anions that occur exclusively in this compound.

**Figure 8:**
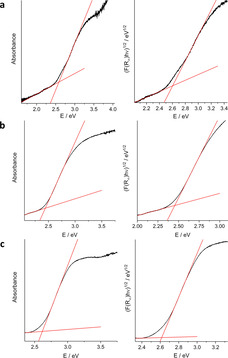
UV‐visible spectra (left hand side) and Tauc plots generated using the Kubelka‐Munk function (*F*(R_∞_)*hν*)^1/*γ*^ with *γ*=0.5 (right hand side) of single‐crystalline **1** (a), **2 a** (b), and **2 b** (c). The measurement was performed under inert conditions employing a Praying Mantis accessory.[^50^, ^51^, ^52^]

As outlined in the introduction, we aimed at affecting not only the development of structural motifs, but also the electronic structures by introducing Sb^III^ into group 14 chalcogenido (semi‐)metallate substructures. To probe the impact on the electronic properties, optical absorption spectra were recorded, which served to illustrate the presence of building units with different elemental compositions, [Ge_2_Sb_2_S_7_]^2−^ and [GeS_4_]^4−^ in **1**, or [Sn_10_O_4_S_20_]^8−^ and [Sb_3_S_6_]^3−^ which are linked by μ‐S‐sharing in **2 a** and **2 b**. Figure 8 summarizes the UV‐visible spectra that were obtained from single crystalline material. The diffuse reflectance spectra (left hand side of Figure 8) were converted to Tauc plots (right hand side of Figure 8) by using the Kubelka‐Munk function, for estimating the nature of the electronic transition. The indicated band gaps (2.62 eV in **1**, 2.44 eV **2 a**, and 2.58 eV in **2 b**) agree well with an allowed indirect transition in the three compounds according to the corresponding Tauc plots (2.61 eV in **1**, 2.45 eV in **2 a**, and 2.62 eV in **2 b**). The allowed direct transitions derived from the Tauc plot (see Figure S5) possess larger energies: 2.78 eV (**1**), 2.74 eV (**2 a**), and 2.80 eV (**2 b**).

We cannot explain the difference of the band gap energies of **2 a** and **2 b** with certainty, but we assume that the highly disordered cations in **2 a** allow for a denser packing of the anions. This is in agreement with a slightly smaller unit cell volume observed for **2 a** (11 098.4(17) Å^3^) as compared to the unit cell in **2 b** (11 431.8(12) Å^3^), see Table S4.

A comparison with the band gaps of the formally underlying binary compounds, GeS_2_ (3.43 eV, direct allowed transition),^[48]^ SnS_2_ (2.38 eV, direct allowed transition),^[6]^ and Sb_2_S_3_ (1.78 eV, direct allowed transition),^[49]^ clearly reflect the multinary elemental composition of the anionic substructures: in **1**, the relatively large amount of Sb^III^ leads to a significant narrowing of the band gap, which is distinctly smaller than that of bulk GeS_2_. The comparably small degree of Sb^III^ incorporation into compound **2**, in contrast, is reflected by a band gap that is relatively close to (a bit larger than) that of SnS_2_—yet one needs to keep in mind that (a) this is a cluster substructure, not bulk SnS_2_, and that (b) the cluster composition is furthermore optically ‘diluted’ by Sn−O units. Beyond this background, a band gap that is similar to the one in SnS_2_ also indicates a notable effect of Sb^III^ incorporation. This is additionally supported by the fact that similarly‐sized crystals of (Cat)_4_[Sn_10_O_4_S_16_(SMe)_4_] possess a lighter yellow color.

## Conclusions

We report about the successful incorporation of (formally) Sb^3+^ ions in anionic substructures of sulfido germanates and sulfido stannates [K_4_(H_2_O)_4_][Ge_4_S_10_] or [K_4_(H_2_O)_4_][SnS_4_], by treatment of these salts with SbCl_3_ in the ionic liquid (C_4_C_1_C_1_Im)[BF_4_], in the ionic liquid mixture (C_4_C_1_C_1_Im)[BF_4_]/(C_4_C_1_C_1_Im)Cl (1:1), or in (C_4_C_1_C_1_Im)Br under ionothermal conditions at 120 °C. The structures of the products, (Cat)_16_[Ge_2_Sb_2_S_7_]_6_[GeS_4_] (**1**) and (Cat)_6_[Sn_10_O_4_S_20_][Sb_3_S_4_]_2_ (**2**), reveal a re‐organization of the original sulfide tetrelate anions. Notably, the [Ge_4_S_10_]^4−^ anion is degraded to form [GeS_4_]^4−^ anions in the substructure of **1**, while the [SnS_4_]^4−^ anions are linked to form supertetrahedral Sn/O/S clusters in the substructure of **2**. Both compounds comprise further anionic subunits involving Sb^III^, with a noradamantane‐type ternary anion in **1** and a cyclic moiety in **2**. The sulfido (semi)metallate units form complex (non‐bonded) assemblies of a total of seven ternary Ge/Sb/S and binary Ge/S anions in **1** and polymeric 1D‐strands of alternating Sn/O/S and Sb/S units in **2**. Owing to a high tendency for disorder of the (C_4_C_1_C_1_Im)^+^ molecules, the counterions could only be located in the crystal structure of **2 b**, comprising (C_4_C_1_C_2_Im)^+^. However, Raman spectra served to corroborate the presence of ionic liquid cations in the other salt. Raman spectra of all three compounds in comparison with that of recently reported (Cat)_4_[Sn_10_O_4_S_16_(SMe)_4_] allowed to assign the most relevant bands. Finally, optical absorption spectra clearly demonstrate the effect of a formal admixture of Sb^III^ to the sulfido (semi)metalate compounds, which leads to significant narrowing of the corresponding band gaps.

## Experimental Section

**General methods**: All reactions and measurements were carried out under dry Ar atmosphere using Schlenk technique or a glovebox (type Unilab plus; MBraun). Elements were used as received: K lumps (Acros Organics, 98 %), Ge powder (ChemPUR, 99.99 %), Sn powder (Sigma–Aldrich, 99 %), and S powder (Alpha Aesar, 99,999 %). Ethylen‐1,2‐diamine (*en*; Sigma–Aldrich, 99,8 %;) was distilled and stored over molecular sieve (3 Å). 1‐Butyl‐2,3‐dimethylimidazolium tetrafluoroborate, (C_4_C_1_C_1_Im)[BF_4_] (Sigma–Aldrich, 98 %), and 1‐butyl‐2,3‐dimethylimidazolium chloride, (C_4_C_1_C_1_Im)Cl (Sigma–Aldrich, ≥97 %), were vacuum‐dried for several days. (C_4_C_1_C_2_Im)Br was synthesized according to a slightly modified literature‐known synthesis procedure.^[53]^ The starting materials K_4_[Ge_4_S_10_] and K_4_[SnS_4_]⋅4 H_2_O were prepared by solid state reaction of K_2_S, Ge or Sn, and S according to the literature.^[38]^ The raw product was purified by water‐extraction.

**Synthesis of 1**: 50 mg of [K_4_(H_2_O)_4_][Ge_4_S_10_] (0.06 mmol, 1.0 equiv), 25 mg of SbCl_3_ (0.11 mmol, 1.8 equiv), 500 mg of (C_4_C_1_C_1_Im)[BF_4_], and 100 μL of ethylene‐1,2‐diamine were filled in a borosilicate glass ampoule, sealed and heated at 120 °C for three days.

**Synthesis of 2 a**: 50 mg of [K_4_(H_2_O)_4_][SnS_4_] (0.11 mmol, 1.0 equiv), 50 mg of SbCl_3_ (0.22 mmol, 2.0 equiv), 250 mg of (C_4_C_1_C_1_Im)[BF_4_], 250 mg of (C_4_C_1_C_1_Im)Cl, and 100 μL of ethylene‐1,2‐diamine were sealed in a borosilicate glass ampoule and heated at 120 °C for three days.

**Synthesis of 2 b**: 50 mg of [K_4_(H_2_O)_4_][SnS_4_] (0.11 mmol, 1.0 equiv), 50 mg of SbCl_3_ (0.22 mmol, 2.0 equiv), 500 mg of (C_4_C_1_C_2_Im)Br, and 100 μL of ethylene‐1,2‐diamine were sealed in a borosilicate glass ampoule and heated at 150 °C for three days.

**Micro‐X ray fluorescence spectroscopy (μ‐XRF)**: Elemental analysis was performed by μ‐XRF with a Bruker M4 Tornado, equipped with an Rh‐target X‐ray tube and a Si drift detector. The emitted fluorescence photons were detected with an acquisition time of 180 s and 240 s. Upon deconvolution of the spectra, quantification of the elements was achieved based on the Ge‐K, S‐K, Sn‐L and Sb‐L, radiation. For μ‐XRF spectra of **1**, **2 a**, and **2 b**, see Figures S2–S4 and Tables S1–S3.

**Single crystal X‐ray diffraction**: X‐ray data was collected on a Stoe StadiVari diffractometer using Cu Kα radiation (*λ*=1.54186 Å; *T*=100 K) equipped with an Oxford Cryosystems module. Structure solution by dual space methods and full‐matrix least‐squares refinement against F2 were carried out using SHELXT15, SHELXL15, and OLEX2 software.^[40]^ The non‐hydrogen atoms were refined using anisotropic displacement parameters. Crystallographic data (excluding structure factors) for the structure reported in this paper have been deposited with the Cambridge Crystallographic Data Centre.

Deposition Numbers 2024786 (**1**), 2024787 (**2 a**), and 2024788 (**2 b**) contain the supplementary crystallographic data for this paper. These data are provided free of charge by the joint Cambridge Crystallographic Data Centre and Fachinformationszentrum Karlsruhe Access Structures service www.ccdc.cam.ac.uk/structures.

**UV‐visible spectroscopy**: Optical absorption spectra were recorded on a Varian Cary 5000/UV/Vis/NIR spectrometer in the range of 200–800 nm in diffuse reflectance mode employing a Praying Mantis^TM^ accessory (Harrick). For ease of viewing, raw data was transformed from %Reflectance R to Absorbance A according to A=log (1/R).^[50]^ The recorded diffuse reflectance spectra were converted in Tauc plots by using the Kubelka‐Munk function (K‐M) to estimate the indicated band gap energies of allowed (in)direct transitions[^51^, ^52^] [Eq. 1]:

(1) $$F\left(R\right)=\frac{k}{s}=\frac{{\left(1-R_{\infty }\right)}^{2}}{{2R}_{\infty }}$$

where *k* is the K‐M absorption coefficient, R∞ is the diffuse reflection, and *s* is the K‐M scattering coefficient.[^54^, ^55^] Tauc plots were generated by plotting ${\left(F\left(R\right)•h\nu \right)}^{1/\gamma }$ as a function of the photon energy *hν*. The power coefficient might be *γ*=1/2 , 2/3 , 2 or 3, depending on the nature of the transition, which corresponds to direct allowed, direct forbidden, indirect allowed, or indirect forbidden transitions, respectively. *E*_g_ is estimated from the intercept with the *x* axis of the linear fit from the corresponding region.^[56]^

**Raman spectroscopy**: Raman data was collected on an S&I MonoVista CRS+ device. The measurements were performed with a laser wavelength of 633 nm and a grating of 300 and 1200 grooves mm^−1^. Each measurement had a duration of 5 s with 10 coadditions and 10 s with 25 coadditions.

## Conflict of interest

The authors declare no conflict of interest.

## Supporting information

As a service to our authors and readers, this journal provides supporting information supplied by the authors. Such materials are peer reviewed and may be re‐organized for online delivery, but are not copy‐edited or typeset. Technical support issues arising from supporting information (other than missing files) should be addressed to the authors.

Supplementary
