# Supplementary material for: Structural Expansion of Chalcogenido Tetrelates in Ionic Liquids by Incorporation of Sulfido Antimonate Units
Source: Chemistry. 2020 Oct 14;26(70):16683–9. doi: 10.1002/chem.202003887 (PMC7756300; doi:10.1002/chem.202003887)
Supplement: Supplementary file 1 — Supplementary [file CHEM-26-16683-s001.pdf]

Chemistry–A European Journal

Supporting Information

Structural Expansion of Chalcogenido Tetrelates in Ionic Liquids by Incorporation of Sulfido Antimonate Units

Bertram Peters, Chloé Krampe, Julian Klärner, and Stefanie Dehnen*[a]

Light Microscopy

Light microscopy investigation of crystals of **2b** (Figure S1) was performed on the stereo light microscope SteREO Discovery.V8 by Carl Zeiss. The microscope was equipped with a high-intensive cold-light source CL 1500 ECO, an Achromat S 0.63x objective (FWD 107 mm), a PL 10x/21 Br ocular, and the microscope camera AxioCam MRc 5 with the camera adapter 60N-C 2/3'' 0,63x. The raw photo material was examined by the AxioVision40x64 4.9.1 SP1 software.

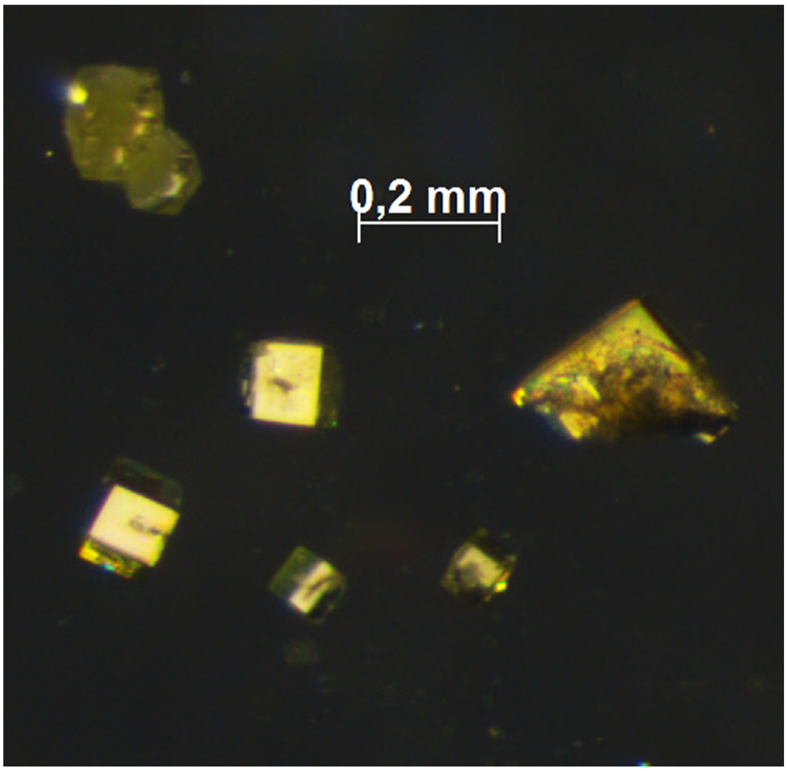

Figure S1. Crystals of **2b** in a light-microscopic image.

Micro-X Ray Fluorescence Spectroscopy (µ-XRF)

Elemental analysis was carried out to investigate the heavy atom ratio of the three compounds and exclude impurities. The chlorine and bromine impurity stem from the ionic liquid. The atom concentrations deviate slightly from the calculated values because of imponderable amounts of immersion-oil residues, which comprise traces of the reactants. Figures S2–S4 and Tables S1–S3 display and summarize the results.

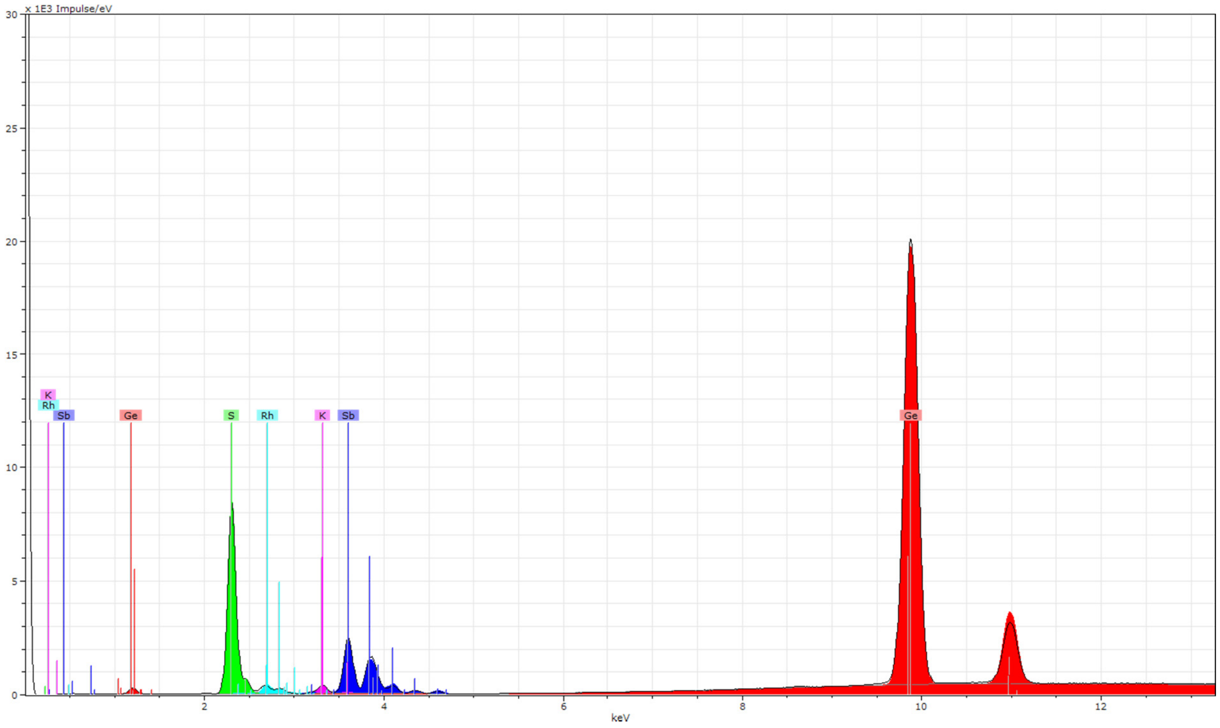

Figure S2. XRF spectra of **1** with polynomial integral fit (S: green; Ge: red; Sb: blue).

Table S1. XRF data of **1**.

Element	X-Ray Series	Norm. Cont. (wt.%)	Atom Cont. (at.%) exp. / calc.	Error (1σ [wt.%])
Ge	K series	20.60	14.31 / 18.3	0.00
S	K series	43.32	68.11 / 64.8	0.03
Sb	L series	33.06	13.69 / 16.9	0.01
K	K series	3.02	3.89 / 0.0	0.00

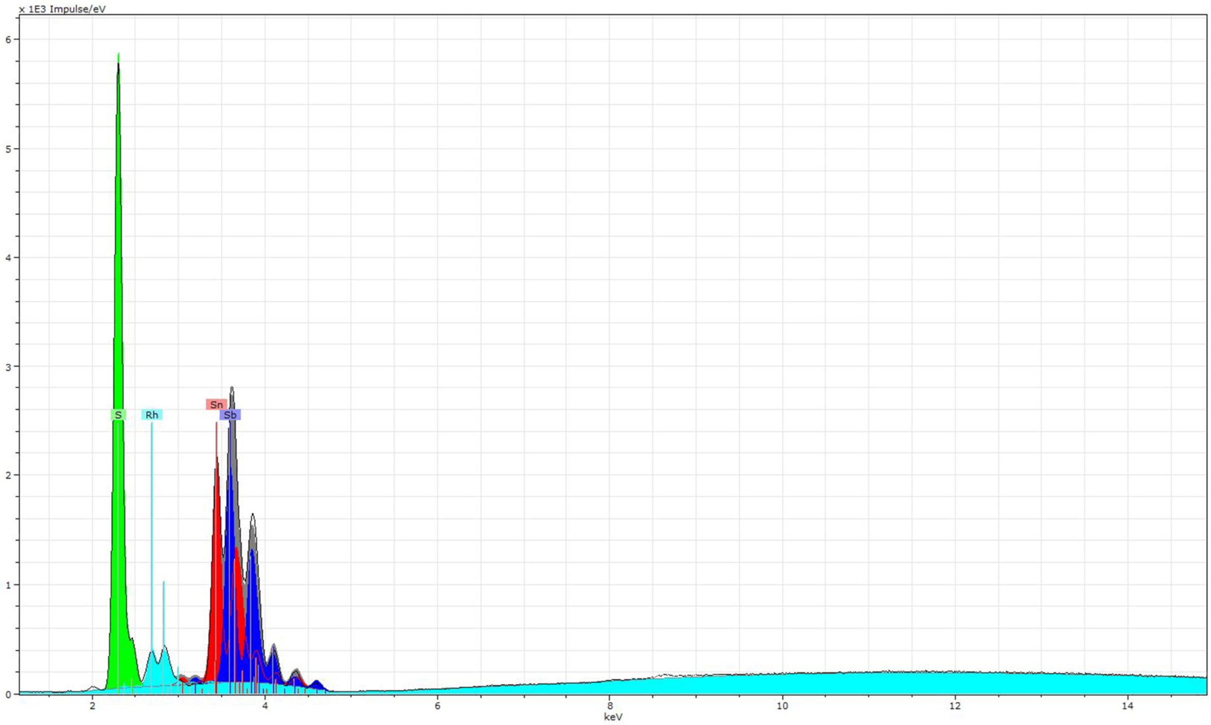

Figure S3. XRF spectra of **2a** with polynomial integral fit (S: green; Sn: red; Sb: blue).

Table S2. XRF data of **2a**.

Element	X-Ray Series	Norm. Cont. (wt.%)	Atom Cont. (at.%) exp. / calc.	Error (1σ [wt.%])
S	K series	30.24	61.89 / 63.6	0.01
Sb	L series	32.41	17.46 / 13.6	0.01
Sn	L series	37.35	20.65 / 22.7	0.01

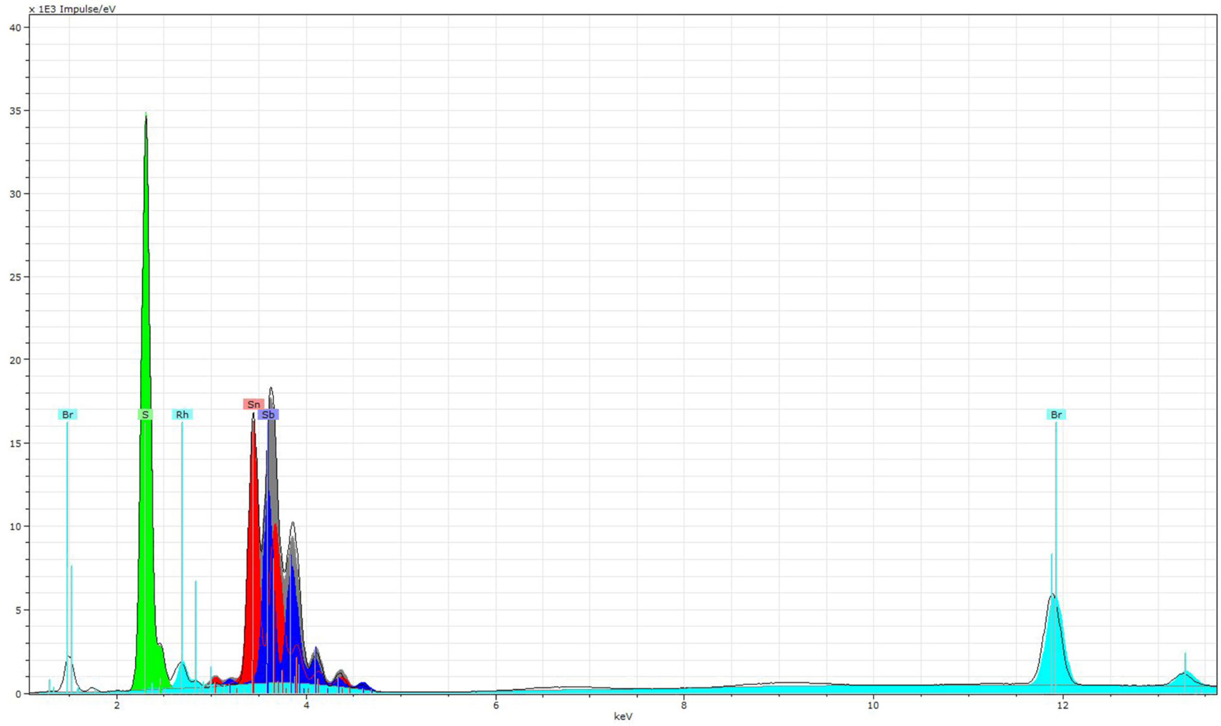

Figure S4. XRF spectra of **2b** with polynomial integral fit (S: green; Sn: red; Sb: blue).

Table S3. XRF data of **2b**.

Element	X-Ray Series	Norm. Cont. (wt.%)	Atom Cont. (at.%) exp. / calc.	Error (1σ [wt.%])
S	K series	25.21	54.84 / 63.6	0.05
Sb	L series	28.27	16.20 / 13.6	0.05
Sn	L series	43.90	25.80 / 22.7	0.07
Br	K series	1.81	1.58 / 0.0	0.00
Cl	K series	0.81	1.59 / 0.0	0.00

Single-Crystal X-Ray Diffraction Studies, Data Collection, Refinement and Crystallographic Details

Crystals suitable for X-ray diffraction analyses were investigated with a StadiVari (**1**, **2a**, **2b**) diffractometer at 100 K. The STOE StadiVari diffractometer used Cu Kα radiation (λ = 1.54186) from an X-ray micro source with X-ray optics and a Pilatus 300K Si hybrid pixel array detector. Upon scaling with spherical absorption correction (STOE X-Area Lana; **1**, **2a**, **2b**), respectively, the structure solution was performed by direct methods, followed by full-matrix-least-squares refinement against *F*2, using SHELXT15, SHELXL15, and OLEX2 software.[1] Table S4 summarizes the crystallographic data for compounds **1**, **2a**, and **2b**.

Comment regarding the *pseudo*-symmetry in 2b versus the missing *pseudo*-symmetry in 2a: Compound **2b** crystallizes in the triclinic space group $P\overline{1}$, although the cell parameters indicate a tetragonal crystal system and the space group $P4_2/mbc$. The latter is due to the fact that the organic constituents of the structure have a low impact only on both the (high) order of the anionic substructures, and on the observed reflections. Consequently, the heavy atoms' symmetry is dominant, and together with pronounced disorder of the cation lead to the observation of a tetragronal *pseudo*-symmetry in **2a**. The cations in **2b** exhibit a significantly lower tendency for disorder and thus possess long-range order. However, their variable orientations still disturb the tetragonal symmetry and lead to an overall triclinic cell. If the organic cations are removed from the density map by application of the back-Fourier-transform method (squeeze),[2], the symmetry of the inorganic framework allows for a structure solution and refinement in the tetragonal crystal system. The refined cell parameters are then a = 19.8273(12) Å, b = 19.8273(12) Å, c = 29.0797(18) Å, α = 90 °, β = 90 °, γ = 90 °.

Table S4. Crystallographic data of **1, 2a**, and **2b**.

Compound (CCDC number)	**1** CCDC-2024786	**2a** CCDC-2024787	**2b** CCDC-2024788
Empirical formula	$Ge_{13}S_{46}Sb_{12}$	$O_4S_{28}Sb_6Sn_{10}$	$C_{43.5}H_{30.25}N_{12}O_4S_{28}Sb_6Sn_{10}$
Formula weight /g·mol^{-1}	3879.43	2879.08	3600.12
Color & shape	yellow blocks	yellow blocks	yellow cubes
Crystal size /mm³	0.053×0.064×0.064	0.046×0.08×0.146	0.083×0.097×0.147
Crystal system, space group	cubic, $Fd\bar{3}m$	tetragonal, $P4_2/mbc$	triclinic, $P\bar{1}$
a /Å	35.3539(16)	19.5455(13)	19.8293(12)
b /Å	35.3539(16)	19.5455(13)	19.8252(12)
c /Å	35.3539(16)	29.051(2)	29.0797(18)
α /°	90	90	89.979(5)
β /°	90	90	90.084(5)
γ /°	90	90	89.989(5)
V /Å³	44189(6)	11098.4(17)	11431.8(12)
Z	8	4	4
ρ_{calc} /g·cm^{-3}	1.166	1.723	2.092
Radiation (λ /Å)	$Cu_{K\alpha}$ (1.54186)	$Cu_{K\alpha}$ (1.54186)	$Cu_{K\alpha}$ (1.54186)
Temperature	100K	100K	100K
μ /mm^{-1}	17.472	33.928	33.149
Min/max transmission	0.0529/0.1528	0.083/0.304	0.085/0.170
F(000)	14112	5144	6645
2θ range /°	8.221–43.378	4.416–76.323	2.697–76.795
No. measured refl.	11907	73651	280080
No. independent refl.	814	5901	46748
No. indep. refl. (I>2σ(I)	528	1440	3194
No. of parameters	36	108	1317
No. of restraints	0	0	0
R(int)	0.1151	0.1570	0.0574
R_1 ($I > 2\theta(I)$)	0.0371	0.0427	0.0447
wR_2 (all data)	0.0973	0.1098	0.1186
S (all data)	0.964	0.616	0.780
$\Delta\rho_{max}$, $\Delta\rho_{min}$ /e·Å^{-3}	0.247/−0.432	0.588/−0.622	1.267/−0.689

UV-Visible Spectra

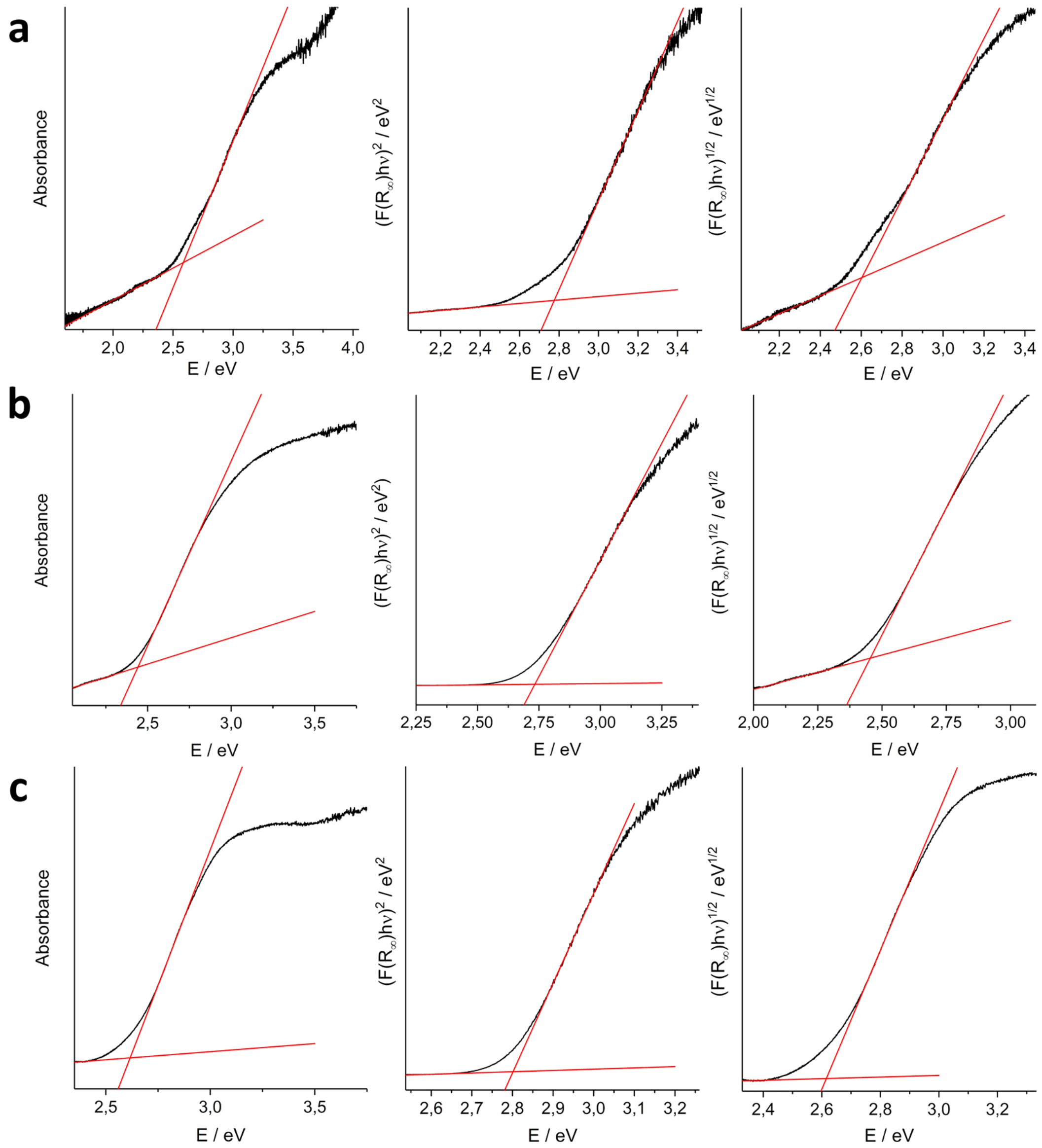

Figure S5. UV-visible spectra (left hand side) and Tauc plots generated using the Kubelka-Munc-function $(F(R_\infty)h\nu)^{1/\gamma}$ (see Methods section in the main document for details)[3-5] with $\gamma = 2$ (center), and $\gamma = 0.5$ (right hand side) of **1** (a), **2a** (b), and **2b** (c). The measurement was performed under inert conditions employing a Praying Mantis accessory.[6-8]

References for the Supporting Information

[1] a) G. M. Sheldrick, *Acta Crystallogr., Sect. A* **2015**, *71*, 3–8; b) G. M. Sheldrick, *Acta Crystallogr., Sect. C* **2015**, *71*, 3–8; c) O. V. Dolomanov, L. J. Bourhis, R. J. Gildea, J. A. K. Howard, H. Puschmann, *J. Appl. Crystallogr.* **2009**, *42*, 339–341.

[2] Spek, A. L., *Acta Crystallogr., Sect. C: Struct. Chem.* **2015**, *71*, 9–18.

[3] P. Kubelka, F. Z. Munk, *Tech. Phys.* **1931**, *12*, 593–601.

[4] A. B. Murphy, *Sol. Energ. Mat. Sol. C.* **2007**, *91*, 1326–1337.

[5] J. Tauc, R. Grigorovici, A. Vancu, *Phys. Stat. Sol.* **1966**, *15*, 627–637.

[6] S. I. Boldish,W. B. White, *Am. Mineral.* **1998**, *83*, 865-871.

[7] A. Escobedo-Morales, I. I. Ruiz-López, M. deL. Ruiz-Peralta, L. Tepech-Carrillo, M. Sánchez-Cantú, J. E. Moreno-Orea, *Heliyon* **2019**, *5*, e01505.

[8] K. A. Michalow, D. Logvinovich, A. Weidenkaff, M. Amberg, G. Fortunato, A. Heel, T. Graule, M. Rekas, *Catal. Today* **2009**, *144*, 7–12.
